# A 3D QSAR Study of Betulinic Acid Derivatives as Anti-Tumor Agents Using Topomer CoMFA: Model Building Studies and Experimental Verification

**DOI:** 10.3390/molecules180910228

**Published:** 2013-08-22

**Authors:** Weimin Ding, Miao Sun, Shaman Luo, Tao Xu, Yibo Cao, Xiufeng Yan, Yang Wang

**Affiliations:** 1 Alkali Soil Natural Environmental Science Center, Northeast Forestry University/Key Laboratory of Saline-Alkali Vegetation Ecology Restoration in Oil Field, Ministry of Education, Harbin 150040, China; E-Mails: dingweimin@hrbust.edu.cn (W.D.); luoshaman@163.com (S.L.); xutao_happy@163.com (T.X.); dingyuhuidebaba@126.com (Y.C.); xfyan@nefu.edu.cn (X.Y.); 2 School of Chemical and Environmental Engineering, Harbin University of Science and Technology, Harbin 150080, China; E-Mail: sunmiao@hrbust.edu.cn

**Keywords:** betulinic acid, betulin, 3D-QSAR, topomer CoMFA, experimental verification

## Abstract

Betulinic acid (BA) is a natural product that exerts its cytotoxicity against various malignant carcinomas without side effects by triggering the mitochondrial pathway to apoptosis. Betulin (BE), the 28-hydroxyl analog of BA, is present in large amounts (up to 30% dry weight) in the outer bark of birch trees, and shares the same pentacyclic triterpenoid core as BA, yet exhibits no significant cytotoxicity. Topomer CoMFA studies were performed on 37 BA and BE derivatives and their *in vitro* anti-cancer activity results (reported as IC_50_ values) against HT29 human colon cancer cells in the present study. All derivatives share a common pentacyclic triterpenoid core and the molecules were split into three pieces by cutting at the C-3 and C-28 sites with a consideration toward structural diversity. The analysis gave a leave-one-out cross-validation q^2^ value of 0.722 and a non-cross-validation r^2^ value of 0.974, which suggested that the model has good predictive ability (q^2^ > 0.2). The contour maps illustrated that bulky and electron-donating groups would be favorable for activity at the C-28 site, and a moderately bulky and electron-withdrawing group near the C-3 site would improve this activity. BE derivatives were designed and synthesized according to the modeling result, whereby bulky electronegative groups (maleyl, phthalyl, and hexahydrophthalyl groups) were directly introduced at the C-28 position of BE. The *in vitro* cytotoxicity values of the given analogs against HT29 cells were consistent with the predicted values, proving that the present topomer CoMFA model is successful and that it could potentially guide the synthesis of new betulinic acid derivatives with high anti-cancer activity. The IC_50_ values of these three new compounds were also assayed in five other tumor cell lines. 28-O-hexahydrophthalyl BE exhibited the greatest anti-cancer activities and its IC_50_ values were lower than those of BA in all cell lines, excluding DU145 cells.

## 1. Introduction

**Figure 1:**
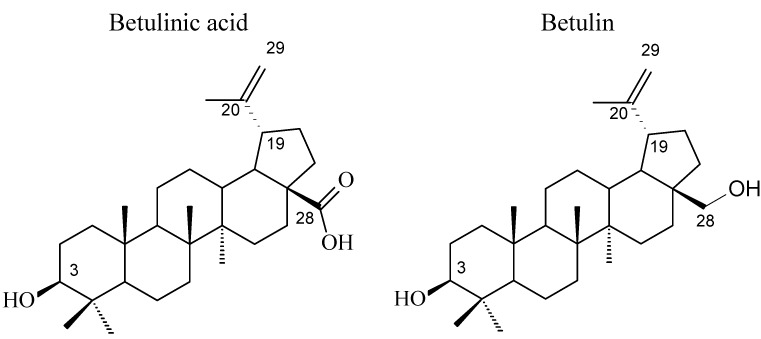
Structures of betulinic acid and betulin.

Betulinic acid (3β-hydroxy-lup-20(29)-en-28-oic acid, BA, Figure 1) is a natural product with notable anti-HIV activity and cytotoxicity against various malignant carcinomas. In particular, BA exerts its cytotoxicity by triggering the mitochondrial pathway to apoptosis [1] without side effects at doses up to 500 mg/kg [2]. Zuco *et al*. [3] reported that BA exhibited selective cytotoxicity for tumor cell lines over normal cells. BA has been suggested for the treatment of human melanoma tumors [4]. Betulin (lup-20(29)-en-3β,28-diol, BE, Figure 1), the 28-hydroxyl analog of BA, was one of the first natural products identified and isolated from plants in 1788 [5], and it is present in large amounts (up to 30% dry weight) in the outer bark of birch trees. In contrast to BA, BE has no significant cytotoxicity. Meanwhile, BE represents an abundant starting material for the production of BA and new anti-HIV and anti-tumor agents. BA derivatives semi-synthesized from BA and BE have been reported, and their anti-tumor bioactivities have been investigated [6,7,8,9,10,11,12,13,14,15,16,17,18]. 

QSAR methods describe structure-activity relationships in terms of physicochemical parameters and steric properties, or certain structural features (Free Wilson analysis) [19,20]. The method of comparative molecular field analysis (CoMFA) was the first real three-dimensional (3D) QSAR method. Although CoMFA is widely succesful in analyzing structure-activity relationships, it still has weaknesses. For example, in the absence of a reliable receptor site, the researcher must choose among a multitude of ligand alignment protocols; meanwhile, only one approach exists for comparing alignment protocols, namely the maximization of various q^2^ (cross-validated r^2^) statistics, which average the internal predictive accuracy for every structure/activity observation. Considering only the measurement uncertainties attached to any individual structure/activity observation, it is doubtful that moderate changes in q^2^ values can provide dependable alignment guidance [21]. This difficulty is addressed with surprisingly good results via a completely objective and universal methodology termed topomer CoMFA, the combination of the universal “topomer” methodology and CoMFA technologies. A series of input structures are broken into two or more fragments at central acyclic single bonds, and any core fragment structurally common to the entire series is removed. Standard topomer 3D models are automatically constructed for each fragment, and a set of steric and electrostatic fields (“CoMFA column”) is generated for each set of topomers [22].

In the present paper, a topomer CoMFA method was utilized to explore the quantitative relationships between some BA derivative structures and their anti-cancer activities. The bioactivities of BA and BE derivatives modified at the C-3 and C-28 sites were compared with the values predicted by topomer CoMFA. Based on this topomer CoMFA analysis, some new molecules were designed and synthesized, and their anti-tumor activities were assessed across five different cancer cell lines, which verified that the topomer CoMFA technology provided useful information for designing new derivatives with enhanced anti-tumor activities.

## 2. Results and Discussion

### 2.1. 3D-QSAR Model using a Topomer CoMFA Method

**Figure 2:**
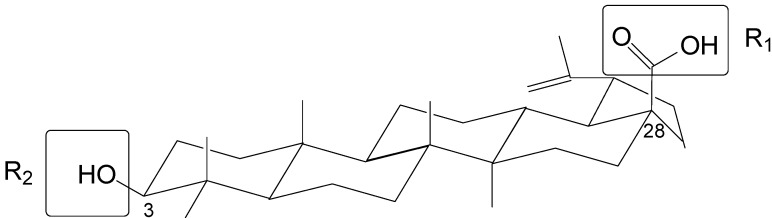
Diagram of split betulinic acid into three pieces for the topomer CoMFA modeling.

The topomer CoMFA method provides a means for an alignment-independent 3D-QSAR approach, which is advantageous because it does not require alignment and it provides a means for automated activity searches in fragment libraries. BA derivatives in the literature have a common pentacyclic triterpenoid core, and most studies have focused on modifications at the C-3 and C-28 sites. In this topomer CoMFA study, we designated the groups at the two sites as R-groups. We split the molecules into three pieces by cutting at the C-3 and C-28 sites with a consideration toward structural diversity (Figure 2). In this study, Gasteiger-Hückel charges were used for charge calculations.

A database alignment was used to establish topomer CoMFA 3D-QSAR models using the methods described in the Experimental Topomer CoMFA setup section. The predicted values of compounds in the training and test sets using topomer CoMFA are listed in Table 1. The experimental IC_50_ value of compound **10** is >100 μM, and compound 10 was selected as a test molecule with the pIC_50_ value set at 4.0 to expand the diversity of structures and the range of pIC_50_. Therefore a bigger residual was found between the predicted and experimental pIC_50_s for compound **10**.

**Table 1:** The experimental pIC_50_s, predicted pIC_50_s (Pred.), and their residuals (Res.) for the training and test set molecules using topomer CoMFA.

Comp.	Substituent		pIC_50_		Res.
	R_1_	R_2_	Experimental	Pred.	
**Test set**					
1	―COOH	―OH	4.856	4.627	0.229
2	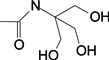	―OCOCH_3_	4.999	4.855	0.144
3	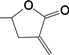	―OCOCH_3_	5.276	5.244	0.032
4	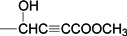	―OH	5.310	5.150	0.160
5	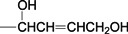	―OCOCH_3_	4.936	5.053	−0.118
6	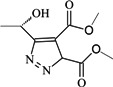	―OCOCH_3_	4.921	4.953	−0.032
7	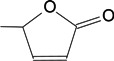	―OH	5.229	4.990	0.239
8	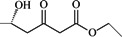	―OCOCH_3_	4.733	4.722	0.011
9	―COOH	―OCONHC_2_H_5_	4.776	4.806	−0.030
10	―COOH	―OCOC_5_H_11_	4.000	4.486	−0.486
11	―CH_2_OCOCH_3_	―OCOCH_3_	4.590	4.782	−0.192
**Training set**					
12	―COOH	―OCOCH_3_	4.792	4.818	−0.026
13	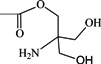	―OCOCH_3_	5.611	5.384	0.227
14	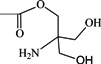	―OH	5.051	5.192	−0.141
15	―CH_2_OCOCH_3_	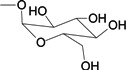	5.009	4.970	0.039
16	―CH_2_OCOCH_3_	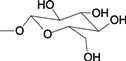	4.997	4.970	0.026
17	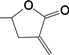	―OH	5.056	5.052	0.004
18	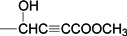	―OCOCH_3_	5.301	5.341	−0.040
19	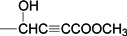	―OCH_3_	5.328	5.334	−0.006
20	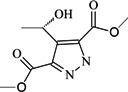	―OCOCH_3_	4.770	4.728	0.042
21	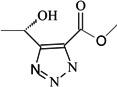	―OCOCH_3_	4.740	4.775	−0.035
22	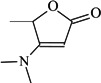	―OCOCH_3_	5.244	5.296	−0.052
23	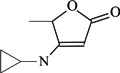	―OCOCH_3_	5.292	5.316	−0.024
24	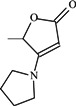	―OCOCH_3_	5.409	5.421	−0.012
25	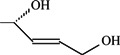	―OCOCH_3_	4.799	4.825	−0.026
26	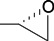	―OH	4.813	4.711	0.102
27	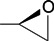	―OH	4.740	4.711	0.029
28	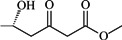	―OCH_3_	4.775	4.715	0.060
29	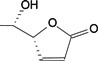	―OCOCH_3_	4.524	4.527	−0.003
30	―COCH_2_COOC_2_H_5_	―OCOCH_3_	4.921	4.914	0.007
31	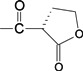	―OCOCH_3_	4.830	4.776	0.054
32	―OCOOCH_3_	―OCONHC_6_H_5_	4.161	4.125	0.036
33	―CH_2_OCONHC_2_H_5_	―OCONHC_2_H_5_	5.208	5.180	0.028
34	―CH_2_OCONHC_2_H_5_	―OCONHC_6_H_5_	4.160	4.176	−0.016
35	―CH_2_OCOCH_3_	―OCONHC_6_H_5_	4.117	4.116	0.001
36	―COOH	―OCOC_9_H_19_	4.279	4.287	−0.008
37	―CH_2_OCOCH_2_CI	―OH	4.559	4.586	−0.027

**Figure 3:**
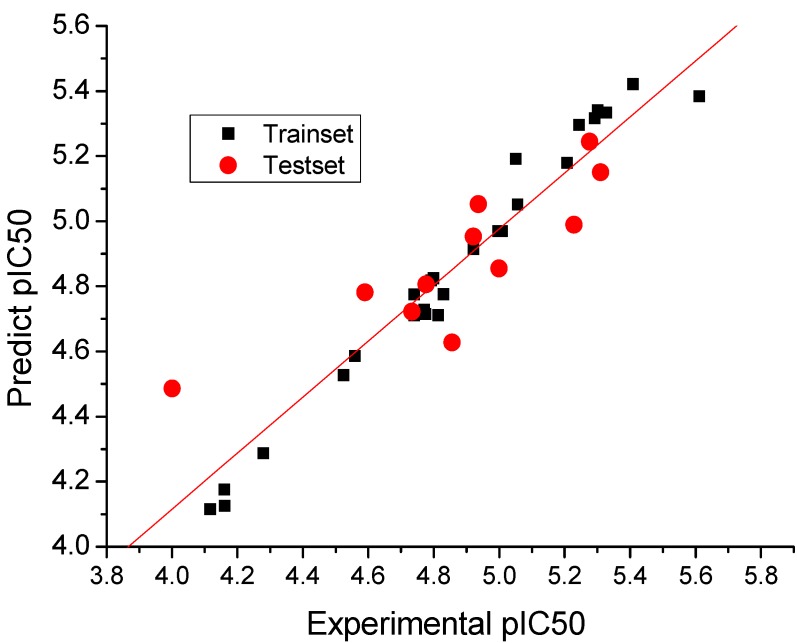
Graph of the experimental versus predicted pIC_50_ values of the training and test set using topomer CoMFA.

The topomer CoMFA model was optimized. Cross-validation q^2^ value of 0.722 and a non-cross-validation r^2^ value of 0.974 with an optimized component of 5 were obtained, which suggested that the model has good predictive ability (q^2^ > 0.5) (F values = 151.669). The predictive correlation coefficient (r^2^_pred_) of 0.675, the R^2^_m_ value of 0.726, and the root mean square error of prediction (RMSEP) value of 0.201 were determined to be externally validated. The pIC_50_ values of the test set were predicted, and the test set r^2^_pred_ value of 0.675 was obtained in the evaluation of the predictive ability of the model. The correlation plot of the experimental versus predicted pIC_50_ values is shown in Figure 3, and a good correlation between the predicted and the experimental values of the compounds was observed. The predictive power of the model is high but only within the addressed range, *i.e.*, truly high-affinity compounds (pIC_50_ > 8) cannot be explained by the model.

**Figure 4:**
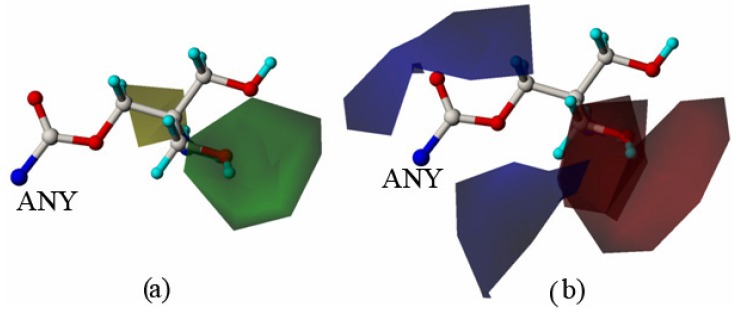
Topomer CoMFA contour maps around R1. (**a**) Steric contour map depicted around R1 (green: favored; yellow disfavored); (**b**) electrostatic contour map depicted around R1. Blue contours indicate the regions where electropositive groups increase activity, whereas red contours indicate the regions where electronegative groups increase activity.

**Figure 5:**
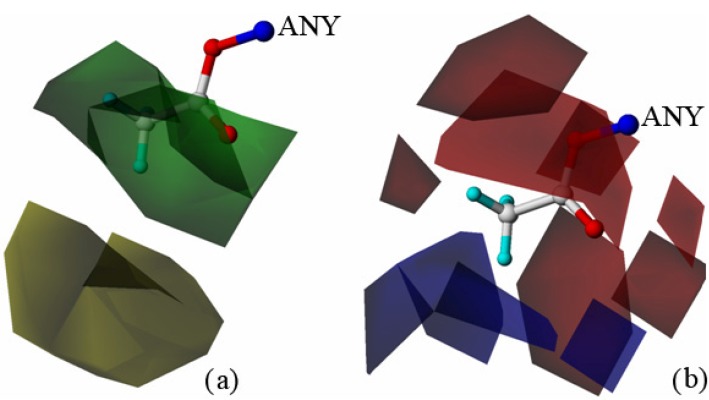
Contour maps of topomer CoMFA around R2. (**a**) Steric contour map depicted around R2 (green favored; yellow disfavored); (**b**) electrostatic contour maps depicted around R2. Blue contours indicate the regions where electropositive groups increase activity, and red contours indicate the regions where electronegative groups increase activity.

The topomer CoMFA 3D contour maps around R1 and R2 (shown in Figure 4 and Figure 5, respectively) were generated by plotting the coefficients from the model. It is better to choose the molecule with highest activity as the reference molecule which makes it is easier to explain the contour map. Compound 13 showed the best biological activity against HT 29 cells in all 37 compounds. Hence these maps are shown using compound 13 as a reference structure. In the steric field, the green contours located at the C-28 site indicate that a bulky substituent would be favorable, and the yellow contours denote where bulky substituents would not be tolerated (Figure 4a). In the electrostatic field, the blue contours located at the C-28 site indicate that electropositive groups would be favorable, and the red contours indicate that electronegative groups would be favorable (Figure 4b). Green contours dominated the R1 group in the steric field, blue contours occupied the middle of the electrostatic field, whereas red contours were located at the end of the substituent; this suggested that bulky groups with electronegative potential at the end of the side chain at the C-28 site would be favorable for activity. Regarding the contours of the R2 group, green contours (Figure 5a) were located near the C-3 site, and yellow contours were located in farther away; the red contours were located in the middle of the electrostatic field, and blue contours were located far away from the C-3 site (Figure 5b); this demonstrated that a moderately bulky group with electronegative potential at the R2 site would improve the anti-tumor activity.

### 2.2. Molecular Design and Experimental Verification

**Scheme 1:**
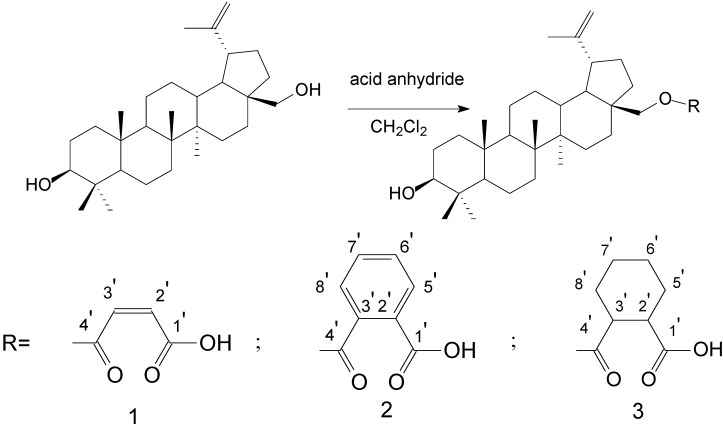
Synthesis of compounds **1**–**3**.

To verify the topomer CoMFA model, molecules were designed. Since BE is the 28-hydroxyl analog of BA and shares the same pentacyclic triterpenoid core with BA, both BA and BE are good starting materials for further derivatization. BE shows better availability and much cheaper price because it is present in large amounts (up to 30% dry weight) in the outer bark of birch trees, so BE was selected as the starting material for the experimental verification. Because a moderately bulky group with electronegative potential was required at the C-3 site, we left this position unchanged, as the hydroxyl group at the C-3 site in BE perfectly matches the requirements. To introduce electronegative potential at the end of the side chain at the C-28 site, we employed dicarboxylic anhydride to form a carboxylic group at the end of side chain via esterification of the C-28 hydroxyl group of BE. To meet the needs of a sterically bulky group, the cyclic dicarboxylic anhydrides phthalic anhydride and hexahydrophthalic anhydride were selected, and maleic anhydride was chosen as a representative fatty acid. The chemical reaction and the substituent groups are illustrated in Scheme 1. The bulky electronegative groups (maleyl, phthalyl, and hexahydrophthalyl groups) were directly introduced at the C-28 position of BE. The anti-cancer activity of these three compounds against HT29 cells were predicted and tested (Table 2). The experimental pIC_50_ were compound **1** < compound **2** < compound **3**, which were consistent with the tendency of predicted values. The residues between the experimental and predicted values were much bigger in Table 2 than that in Table 1, because the experimental IC50 results from our lab may be significantly different from Kommera’s lab, which is also the reason we should select the data from the same research group to establish a reliable model. The consistent tendency further confirmed the established topomer CoMFA method as a predictive model for the structure-activity relationships of BA derivatives. The hexahydrophthalyl and phthalyl derivatives displayed better or equal bioactivity to BA, which is much more active than the parent molecule BE.

**Table 2:** The predicted and experimental activity against HT29 cell line.

Compd.	IC_50_ (μM)	pIC_50−_ (Exp.)	pIC_50_ (Pred.)
**1**	83.09 ± 7.53	4.080	4.925
**2**	35.33 ± 1.04	4.452	5.071
**3**	30.05 ± 1.54	4.522	5.127
BA	32.66 ± 0.62	4.486	4.627
	13.93 ± 0.46 *	4.856 *	

* data from literature [11,12,13].

To better understand the anti-tumor activities of these three derivatives, their IC_50_ values were also assayed in five other tumor cell lines, including pancreatic cancer (MPC2), prostate cancer (DU145), lung carcinoma (NCI-H520), cervical cancer (HeLa), and ovarian cancer (2774) cells. The results are presented in Table 3. Among these three structures, compound 3 (containing the hexahydrophthalyl group) exhibited the greatest anti-cancer activities; its IC_50_ values were lower than those of BA in all cell lines excluding DU145 cells. Following the instructions of the topomer CoMFA model, additional molecular design can be conducted, and more promising and potent agents can be synthesized.

**Table 3:** Cytotoxicity of the compounds against a panel of human cancer cell lines.

Compd.	IC_50_ (μM) for cancer cell lines				
	MPC2 (pancreatic cancer)	DU145 (prostate cancer)	NCI-H520 (lung carcinoma)	HeLa (cervical cancer)	2774 (ovarian cancer)
BA	38.58 ± 2.91	23.27 ± 2.20	19.60 ± 2.44	25.93 ± 1.87	39.54 ± 2.19
**1**	75.07 ± 4.96	92.54 ± 1.45	84.83 ± 2.15	99.62 ± 4.64	67.93 ± 2.00
**2**	31.28 ± 3.43	36.19 ± 2.77	31.24 ± 0.46	40.05 ± 3.37	27.02 ± 2.92
**3**	22.79 ± 0.35	37.99 ± 2.50	18.55 ± 0.38	17.47 ± 1.48	25.85 ± 1.34

## 3. Experimental

### 3.1. Data Sets

The *in vitro* anti-cancer activities of 37 BA and BE derivatives (reported as IC_50_s) against HT29 human colon cancer cells were used for the present study. All compounds and associated inhibitory activity data were obtained from the literature [10,11,12,13] reported by the same research group within 2 years. These 37 molecules were divided into a training set of 26 compounds and a test set of 11 compounds. IC_50_ values were converted to pIC_50_ values by taking log (1/IC_50_). The compounds in the test set were randomly selected with the goal of choosing a range of both biological activity data and structural diversity. The structures of the compounds in the training and test sets are listed in Table 1.

### 3.2. Topomer CoMFA Setup

The Topomer CoMFA program in sybyl-x1.1 software package was used to perform all calculations. Topomer CoMFA is an alignment-independent 3D-QSAR method that combines the topomer search method [23] (a fragment alignment approach) with the conventional CoMFA method. A 3D-QSAR model was generated by splitting the molecules into fragments, topomerically aligning each fragment, and calculating steric and electrostatic field descriptor values for the topomerically aligned fragments to create a CoMFA table with the field descriptor values. Besides the core of the molecule, we split side functional group into two R-groups that refer to the R1 and R2 groups of BA (Figure 2).

To evaluate the predictive ability of the model, the pIC_50_ values of the test set were predicted using the model developed using the training set. The optimization and splitting methods of the test set were the same as those described for the training set.

To evaluate the predictive ability of the model, structure optimization of the test set was performed as previously described for the training set. The pIC_50_ values of the test set were predicted on the basis of the constructed model. r^2^_pred_, which measures the predictive performance of a PLS model, is defined by equation 1 as follows:

r^2^_pred_ = (SD − PRESS)/SD (1)

where SD is the sum of the squared deviations between the predicted biological activities of the test set and the mean activities of the training molecules and PRESS is the sum of the squared deviations between the predicted and experimental activity values for every molecule in the test set.

Furthermore, the r^2^ value between the observed and predicted activity values of the compounds in the test set with (r^2^) or without an intercept (r^2^_0_) and the RMSEP [defined by Equation (2)] [24] for the test set compounds were also calculated. The models were validated using an additional parameter, R^2^_m_ [defined by Equation (3)], which is a good indicator of the external predictability of QSAR models [25]:

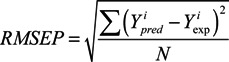
(2)

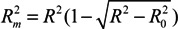
(3)


### 3.3. Materials and Instruments

BE was extracted from the bark of *Betula platyphylla* Suk., and its purity exceeded 95% as determined by HPLC. All other chemicals (reagent grade) were commercially available. Separation of compounds **1**–**3** was performed by column chromatography using silica gel 60 (70–230 mesh). Thin-layer chromatography was performed using silica gel-coated aluminum sheets (silica gel 60 GF254, E. Merck, Darmstadt, Germany). The melting point (m.p.) of each compound measured on an X-6 micro melting-point apparatus (Beijing Tech instrument Co., Beijing, China). The FTIR spectra were recorded using a Nicolet Mattson Infinity Gold FTIR spectrometer using KBr pellets. ^1^H-NMR spectra (300 MHz) and ^13^C-NMR spectra (75 MHz) were recorded in CDCl_3_ at 25 °C with TMS and solvent signals used as internal standards using a Bruker AVANCE-300 MHz. Chemical shifts are reported in ppm (δ). Mass spectra were obtained using a Finnigan Mat 95 apparatus. Elemental analyses were performed on a Thermo Finnigan EA 1112 Series Flash Elemental Analyzer, and the obtained values were within 0.4% of the theoretical values. 

### 3.4. General Procedure for Synthesizing BE Esters

BE (1 mmol) and acid anhydride (1 mmol) were refluxed in methylene dichloride (50 mL) under stirring for 30 hours. After completion of the reaction, the reactant was washed with saturated sodium bicarbonate and brine solutions, dried over anhydrous magnesium sulfate, filtered, and evaporated *in vacuo*. The product was purified by column chromatography with a mobile phase of ethyl acetate/petroleum ether (1/3). 

#### 3.4.1. BE 28-*O*-Maleate **1**

White solid, yield 61.0%. m.p. 249–252 °C IR (film) ν_max_: 3407, 2933, 2868, 1717, 1640, 1454, 1250, 1224, 1159, 994 cm^−1^. ^1^H-NMR (CDCl_3_, δ ppm): 6.50 (d, *J* = 12.8 Hz, 1H, H-2′), 6.41 (d, *J* = 12.8 Hz, 1H, H-3′), 4.71 (s, 1H, H-29_a_), 4.62 (s, 1H, H-29_b_), 4.52 (d, *J* = 11.1 Hz, 1H, H-28_a_), 4.08 (d, *J* = 11.3 Hz, 1H, H-28_b_), 3.22 (dd, *J* = 10.8 Hz, *J* = 5.3 Hz 1H, H-3), 2.45 (dt, 1H, H-19). ^13^C-NMR (δ ppm): 168.6 (C1′), 164.0 (C4′), 149.6 (C20), 137.1 (C3′), 129.1 (C2′), 110.2 (C29), 79.0 (C3), 65.9 (C28). MS (ESI, MeOH): *m/z* = 539.3 [M−1]^−^. Anal. Calcd for C_34_H_5__2_O_5_: C, 75.52; H, 9.69. Found: C, 75.35; H, 9.81.

#### 3.4.2. BE 28-*O*-Phthalate **2**

White solid, yield 56.7%. m.p. 193–196 °C. IR (film) ν_max_: 3420, 2921, 1716, 1638, 1452, 1262, 1078, 880, 792 cm^−1^. ^1^H-NMR (CDCl_3_, δ ppm): 7.92 (d, *J* = 2.1 Hz, 1H, H-5′), 7.74 (d, *J* = 5.4 Hz, 1H, H-8′), 7.62–7.57 (dt, 2H, H-6′; H-7′), 4.72 (s, 1H, H-29_a_), 4.61 (s, 1H, H-29_b_), 4.53 (d, *J* = 9.9 Hz, 1H, H-28_b_), 4.15 (d, *J* = 11.1 Hz, 1H, H-28_b_), 3.22 (dd, *J* = 10.8 Hz, *J* = 5.1 Hz, 1H, H-3), 2.54 (dt, *J* = 10.8 Hz, *J* = 5.7 Hz, 1H, H-19); ^13^C-NMR (CDCl_3_, δ ppm): 170.9 (C1′), 168.7 (C4′), 150.2 (C20), 133.3 (C7′), 132.1 (C6′), 130.9 (C2′; C3′), 130.2 (C5′), 129.0 (C8′) ,109.8 (C29), 79.1 (C3), 64.6 (C28); MS (ESI, MeOH): *m/z* = 589.7 [M−1]^−^. Anal. Calcd for C_38_H_54_O_5_: C, 77.25; H, 9.21. Found: C, 77.67; H, 9.50.

#### 3.4.3. BE 28-*O*-Hexahydrophthalate **3**

White solid: Yield 71.0%. m.p. 216–219 °C. IR (film) ν_max_: 3226, 2942, 2868, 1726, 1641, 1705, 1452, 1245, 1221, 1182 cm^−1^. ^1^H-NMR (CDCl_3_, δ ppm): 4.68 (s, 1H, H-29_a_), 4.58 (s, 1H, H-29_b_), 4.33 (dt, *J* = 31.5 Hz, *J* = 9.0 Hz, 1H, H-28_a_), 3.89 (dt, 1H, H-28_b_), 3.18 (dd, 1H, H-3), 2.68–2.63 (dt, 2H, H-2′; H-3′), 2.05 (dt, 1H, H-19); ^13^C-NMR (CDCl_3_, δ ppm): 179.6 (C1′), 173.9 (C4′), 150.2 (C20), 109.8 (C29), 79.1 (C3), 63.0 (C28). MS (ESI, MeOH): *m/z* = 595.4 [M−1]^−^ Anal. Calcd for C_38_H_60_O_5_: C, 76.47; H, 10.13. Found: C, 76.14; H, 10.06.

### 3.5. Cell Culture and *in Vitro* Cytotoxicity Assay

HT29, MPC2, DU145, NCI-H520, HeLa, and 2774 cells were cultured in RPMI-1640 medium (GIBCO, CA, USA) supplemented with 10% fetal bovine serum (FBS; PAA, Yeovil, UK), l-glutamine (0.29 mg/mL), and 1% penicillin-streptomycin in a humidified atmosphere of 5% CO_2_ at 37 °C. Cells were sub-cultured every 4–5 days by total replacement using 0.25% (w/v) trypsin. *In vitro* cytotoxicity assays were performed according to a previously reported method [26,27]. Briefly, cells were seeded in 96-well culture plates in 0.2 mL of growth medium per well and allowed to attach for 24 h. The culture medium was replaced with either BA or the derivatives at different concentrations in 4 replicates followed by 72 h of incubation. After incubation, 20 μL of 3-(4,5-dimethylthiazol-2-yl)-2,5-diphenyl tetrazolium bromide (MTT; Amresco, Solon, OH, USA) solution at a concentration of 5 mg/mL was added to each well followed by incubation for 4 h. The MTT solution was then aspirated, and 150 μL of DMSO was added to each well to dissolve the dark blue crystals thoroughly. The absorbance was measured at 490 nm using a microplate reader (Infinite 200 NanoQuant, Tecan, Grödig, Austria). The relative growth rate (%) was calculated as (mean absorbance of the sample/mean absorbance of the control) × 100%, considering the optical density of the control as 100%.

## 4. Conclusions

Topomer CoMFA studies were performed on 37 BA derivatives. The analysis gave a leave-one-out cross-validation q^2^ value of 0.722 and a non-cross-validation r^2^ value of 0.974, which suggested that the model has good predictive ability (q^2^ > 0.2). The contour maps illustrated that bulky and electron-donating groups would be favorable for activity at the C-28 site and that a moderately bulky and electron-withdrawing group near the C-3 site would improve the activity for the R2 group. According to the modeling result, three betulin derivatives **1**–**3** were designed and synthesized. The *in vitro* cytotoxicity of the given analogs was consistent with the predicted values, which proves that the present topomer CoMFA model is successful and that it could guide the synthesis of new BA derivatives with high anti-cancer activity.
